# Intrinsic Disorder-Based Emergence in Cellular Biology: Physiological and Pathological Liquid-Liquid Phase Transitions in Cells

**DOI:** 10.3390/polym11060990

**Published:** 2019-06-04

**Authors:** April L. Darling, Boris Y. Zaslavsky, Vladimir N. Uversky

**Affiliations:** 1 Department of Molecular Medicine and Byrd Alzheimer’s Research Institute, Morsani College of Medicine, University of South Florida, Tampa, FL 33612, USA; aldarlin@mail.usf.edu; 2 Cleveland Diagnostics, 3615 Superior Ave., Suite 4407B, Cleveland, OH 44114, USA; Boris.Zaslavsky@Cleveland-Diagnostics.com; 3 Institute for Biological Instrumentation of the Russian Academy of Sciences, Pushchino, Moscow region 142290, Russia

**Keywords:** intrinsically disordered protein, intrinsically disordered protein region, liquid-liquid phase transition, protein-protein interaction, protein-nucleic acid interaction, posttranslational modifications

## Abstract

The visible outcome of liquid-liquid phase transitions (LLPTs) in cells is the formation and disintegration of various proteinaceous membrane-less organelles (PMLOs). Although LLPTs and related PMLOs have been observed in living cells for over 200 years, the physiological functions of these transitions (also known as liquid-liquid phase separation, LLPS) are just starting to be understood. While unveiling the functionality of these transitions is important, they have come into light more recently due to the association of abnormal LLPTs with various pathological conditions. In fact, several maladies, such as various cancers, different neurodegenerative diseases, and cardiovascular diseases, are known to be associated with either aberrant LLPTs or some pathological transformations within the resultant PMLOs. Here, we will highlight both the physiological functions of cellular liquid-liquid phase transitions as well as the pathological consequences produced through both dysregulated biogenesis of PMLOs and the loss of their dynamics. We will also discuss the potential downstream toxic effects of proteins that are involved in pathological formations.

## 1. Intrinsically Disordered Proteins

There are no doubts that many biological functions do not require unique protein structures. As a result, not all functional proteins are structured throughout their entire lengths, with many of them being highly flexible in their entire length or containing long structurally disordered regions [1,2,3,4,5,6,7,8,9,10,11,12]. Although the existence and wide commonness of such “floppy” proteins were originally taken with a grain of salt, multiple bioinformatics studies unequivocally revealed that intrinsically disordered proteins (IDPs) and hybrid proteins with ordered domains and intrinsically disordered protein regions (IDPRs) are highly abundant in nature [13,14,15,16,17], with ~25–30% of eukaryotic proteins being mostly disordered [15], more than half of eukaryotic proteins having long regions of disorder [13,14,15], and >70% of signaling proteins possessing long disordered regions [18]. The functions of IDPs and IDPRs are typically complementary to functions of ordered proteins and domains [1,3,5,9,19].

Instead of possessing unique three-dimensional (3-D) structures, these IDPs and IDPRs exist as highly dynamic conformational ensembles of rapidly interconverting structurally different species (or highly dynamic sets of short-lived structures) either at the secondary or tertiary structure levels, and, on the whole protein scale, can be present as collapsed (molten globule-like) or extended (coil- or pre-molten globule-like) forms [2,4,6,7,20,21]. One should keep in mind though that although a protein molecule is often considered as a semi-homogeneous entity, which is either entirely ordered or disordered, or consisting of homogeneously ordered and/or disordered regions/domains, this picture represents an obvious oversimplification. In fact, the structures of many proteins represent an ensemble of differently folded regions with different levels of conformational stability, and different degrees of intrinsic disorderedness. It was pointed out that at the basic level, the inability of IDPs/IDPRs to have unique 3-D-structures is determined by their amino acid sequences, characterized by the presence of compositional biases (depletion in order-promoting residues Trp, Tyr, Phe, Ile, Leu, Val, Cys, and Asn and enrichment in disorder-promoting residues Ala, Arg, Gly, Gln, Ser, Glu, Lys, and Pro), low sequence complexity, presence of repeats, low overall hydrophobicity, high net charge, and many other features [3,22,23,24,25]. However, this inability to fold is unequally distributed within an amino acid sequence. As a result, different parts of a protein molecule can be under-folded to different degrees. This defines an astonishing multi-level spatiotemporal heterogeneity of IDP/IDPR, whose mosaic structure represents a complex combination of foldons (independently foldable units), inducible foldons (disordered regions that can (partially) fold at interaction with the binding partners), morphing inducible foldons (disordered regions that can differently fold at interaction with different binding partners), non-foldons (non-foldable protein regions), semi-foldons (regions that are always in a semi-folded form), and unfoldons (ordered regions that have to undergo an order-to-disorder transition to become functional) [21,26,27,28,29]. One should also keep in mind that it is extremely unlikely that different foldons in a given protein would possess identical conformational stability. Furthermore, foldons are known to continually unfold and refold even under native conditions [30,31,32]. As a result, at any given moment, even a well-folded, ordered protein would have a mosaic structure, possessing a set of temporary folded and unfolded foldons [29].

Despite an obvious structure-function paradigm-based expectation that structural ‘floppiness’ would be incompatible with protein functionality, numerous protein functions are derived from the lack of ordered structure in a protein molecule. In fact, IDPs/IDPRs can be grouped functionally into several broad classes, such as molecular recognition, molecular assembly, protein modification, entropic chain activities, and RNA and protein chaperones [33,34]. Furthermore, structural ‘floppiness’ also defines the ability of IDP/IDPR to be controlled and regulated at multiple levels [21,26,35,36,37], with various post-translational modifications (PTMs) being one of the most important means of such disorder-centered regulation [38,39].

Among various function-related peculiairities of IDPs/IDPRs are their binding promiscuity and crucial involvement in functions related to regulation and control of various cellular processes [2,3,6,7,20,21,33,35,36,40]. The aforementioned binding promiscuity of IDPs/IDPRs is directly related to their recognition functions that can be realized via several molecular mechanisms typically associated with the appearance of specific bound conformations, which are either a result from the binding-induced disorder-to-order transitions promoted in IDPs/IRDs by their partners in a template-dependent manner or are selected by a partner from the conformational ensemble of the corresponding IDP/IDPR. For example, one of the modes of the disorder-based binding represents Molecular Recognition Feature (MoRF) model involving a short foldable binding region located within a longer disordered region [41,42,43,44]. Alternative and complementary modes of the MoRF-like interactions are given by the Short Linear Motif (SLiM) or Eukaryotic Linear Motif (ELM) models that use specific sequence motifs recognizable by binding partners [45]. In the ANCHOR model of the disorder-based binding, IDPs/IDPRs contain segments that are likely to fold in conjunction with a globular binding partner [46,47]. In the primary contact site (PCS) model, certain regions within the disordered ensemble are more exposed than others, and thereby may serve as the first contact sites with the partner [48]. It was also indicated that the unbound state of some IDPs/IDPRs might possess a strong conformational inclination towards their bound structures thereby; i.e., they use partially/transiently pre-formed elements for recognition [49]. In other words, although IDPs/IDPRs lack the hydrophobic cores typical for ordered proteins and do not have unique 3-D-structures, many of them are not completely structure-less random coils, but have some local preferences for transient secondary structure elements and even for some transient tertiary contacts. Such dynamic pre-organization imposes spatial restrictions on IDPs, therefore exposing some of their potential contact sites. The existence of such pre-formed binding sites enables faster and more effective interactions of IDPs with their targets [9,41,49,50].

Many IDPs/IDPRs are characterized by multifunctionality, which is commonly found ‘moonlighting’ proteins [51], and which is determined by the mosaic architecture of IDPs/IDPRs with multiple relatively short and differently folded functional elements spread within the amino acid sequences [26]. Furthermore, IDPs and hybrid proteins with IDPRs can be specifically compartmentalized within a cell, being responsible for the biogenesis of different proteinaceous membrane-less organelles (PMLOs) [52,53,54,55,56], which represent a crucial illustration of the emergent behavior of IDPs/IDPRs related to their “edge of chaos” character.

## 2. Intrinsically Disordered Proteins as “Edge of Chaos” Systems

It was pointed out that IDPs/IDPRs can be considered as the “edge of chaos” systems operating in a region located between order and chaos (complete randomness) and characterized by maximal complexity [26,57,58]. Such positioning of IDPs/IDPRs at the “edge of chaos” has multiple important consequences, such as their exceptional structural and functional heterogeneity and their extreme sensitivity to small changes in the environment that can generate large and diversified changes and that represents an important molecular mechanism of the exquisite functional and structural control of IDPs/IDPRs by a variety of environmental means [21,26,35,36,37,57,58,59].

In general, complex or edge of chaos systems are known to be characterized by a set of specific features, such as [60]:

(i) Presence of many heterogeneous components involved in nonlinear interactions. As a result, behavior of such systems cannot be described as a simple sum or multiples of the behaviors of their parts. Furthermore, a small perturbation may cause a large effect, a proportional effect, or even no effect at all;
(ii) Interdependence of the constituents of a complex system;
(iii) Complex structure spanning several scales, with the components of a complex system being complex systems themselves;
(iv) The presence of emergent, unanticipated behavior, such as the arising of novel and coherent structures, patterns, and properties during the process of self-organization;
(v) A constant interplay between chaos (disorder) and order;
(vi) Important interrelations between competition and cooperation, generating both positive (amplifying) and negative (damping) feedbacks;
(vii) The presence of a memory, where the history of a complex system (i.e., its prior states) is important for its present and future states.

All these features and their various combinations can be found in IDPs/IDPRs [26,57,58], which are characterized by exceptional spatiotemporal complexity and structural heterogeneity. In fact, IDPs/IDPRs are heterogeneous at multiple levels, being, globally, compact or extended to different degree, and also containing heterogeneous structural constituent (foldons, induced foldons, semi-foldons, non-foldons, and unfoldons), which can be independent or interdependent and can interact nonlinearly. These differently (dis)ordered structural components are always moving between order and disorder. Due to their complex structural organization and ability to undergo fast structural changes IDPs/IDPRs possess high environmental sensitivity and responsiveness, being able to sense different stimuli and response to them via corresponding structural changes. The memory of IDPs/IDPRs is defined by the existence of MoRFs, SLiMs, and PreSMos, which are transiently populated in the non-bound state and may have a profound influence on the binding mechanism and on the resulting bound state of an IDP/IDPR. Finally, IDPs/IDPRs possess emergent behavior, since under some conditions they undergo self-organization via stimuli-induced disorder-to-order transitions. Even more impressively, self-organization of IDPs/IDPRs can generate unanticipated novel structures, patterns, and properties [26]. Among related phenomena are oscillatory self-organized emergent behavior of some bacterial systems, as well as liquid-liquid phase separation-driven formation of various proteinaceous membrane-less organelles (PMLOs), which are commonly found in both prokaryotic and eukaryotic cells, formation of large protein clusters on the membrane surface, and liquid-gel phase transitions. Some of the outputs of such emergent behavior of IDPs/IDPRs are considered below.

## 3. Emergent Behavior of IDPs/IDPRs

### 3.1. Some Biophysics Behind the Disorder-Driven Liquid-Liquid Phase Transitions

**Figure 1:**
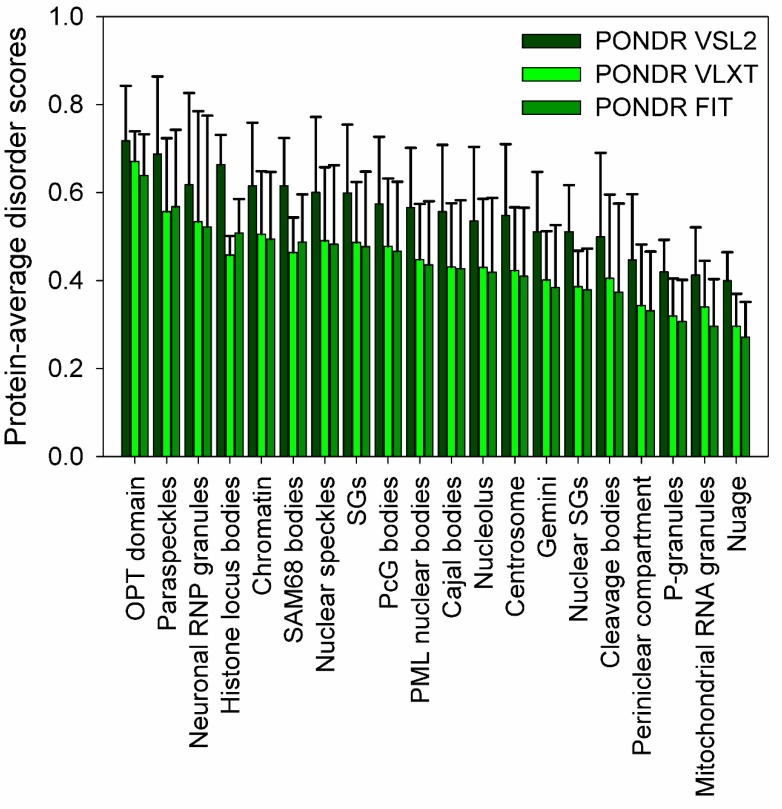
Intrinsic disorder status of proteinaceous membrane-less organelles (PMLO)-related proteins. Evaluation of the overall disorder levels in human proteins associated with PMLOs. Spread of the protein-average disorder scores in individual PMLOs evaluated by PONDR^®^ VSL2 (black bars), PONDR^®^ VLXT (red bars) and PONDR^®^ FIT (green bars) is shown. Bars represent mean protein-average disorder scores in corresponding PMLOs, whereas error bars reflect the corresponding standard deviations. This image was generated using data presented in ref. [53].

Recent studies clearly indicated that cytoplasm, mitochondria, chloroplasts, and nucleus of eukaryotic cells, as well as cytoplasm of bacterial cells, contain numerous PMLOs. Although typical PMLO contains protein molecules as well as RNA (and/or DNA) [61], contents and compositions of only a few PMLOs partially overlap. Furthermore, not all proteins are capable of LLPTs (at least under the physiological conditions). Therefore, an important question is related to biophysical properties that define the capability to proteins to undergo LLPT and orchestrate assembly and disassembly of very different PMLOs located in very different parts of the cell. It was shown for some eukaryotic PMLOs, such as nuages [62], nucleolus [63], P-granules [64], and RNA granules [65], computationally validated for several nuclear PMLOs [52], hypothesized for some macromolecular “assemblages” [66,67], and generalized for all PMLOs and complex biological coacervates [55,56,68] that their formation might be critically dependent on specific IDPs. More generally, recent studies showed that the proteins that drive LLPTs are often either IDPs of hybrid proteins with IDRPs containing low complexity domains (LCDs) consisting of repeat amino acids with low diversity favoring polar and charged groups [56,69,70]. This conclusion is illustrated by Figure 1, which represents the results of the comprehensive bioinformatics analysis of the proteomes of several PMLOs and shows that PMLO-related proteins contain high levels of intrinsic disorder [53]. Lack of structure in IDPs or hybrid proteins containing IDPRs and ordered domains is extremely important for the PMLO formation and maintenance for many reasons. These proteins have a conformational flexibility that allows for the fluidity of the organelle, are able to form a multitude of weak multivalent transient contacts [29,55,56], which adds to the stability of the PMLO [53]. These weak multivalent interactions range from the heterologous electrostatic attraction between the oppositely charged biological polymers, such as oppositely charged proteins or positively charged proteins and nucleic acids, to homologous interactions of the same protein molecules containing repetitive donor and acceptor domains/regions (e.g., multiple stretches of positively- and negatively-charged residues) needed for the multivalent binding [55,56].

Since many of the PMLO resident proteins are IDPs, and since formation of all the PMLOs analyzed thus far relies on IDPs/IDPRs, it is clear that intrinsic disorder is crucial for the PMLO biogenesis [68]. In other words, PMLOs represent an intricate form of the disorder-based protein complexes [29,55,56], which serve as important illustrations of emerging behavior, and which are highly dynamic in nature and can be formed without noticeable structural changes in the proteins undergoing LLPTs [62]. The structural integrity and biogenesis of PMLOs are both exclusively determined by protein–protein, protein–RNA, and/or protein–DNA interactions [71,72], and the process of PMLO formation is highly controlled, completely reversible, and strongly condition-dependent [55,56]. These PMLOs arise through phase separation of their components and mediate subcellular organization of macromolecules within unique microenvironments [73,74].

Important questions are what makes IDPs/IDPRs the most suitable candidates for biological LLPTs and what defines their roles in regulation and control of the formation and disassembly of various PMLOs. Among the obvious answers to this question are the overall high abundance of IDPs/IDPRs in eukaryotic cells, their lack of fixed structure, and the well-known ability of these proteins to be involved in a wide spectrum of interactions of different physico-chemical nature. In fact, phase separation leading to the PMLO formation is driven partially by weak, multivalent interactions (i.e., electrostatic, π-π, cation-π) [75] between one or more IDPs/IDPRs and (not always) nucleic acids. Some of the biophysical properties of IDPs/IDPRs related to their capability to undergo LLPTs and control biogenesis of PMLOs are outlined below.

(1) Since IDPs/IDPRs typically contain a high number of charged residues being depleted in hydrophobic residues, it is expected that electrostatic interactions would play an important role in conformational behavior and interactability of IDPs/IDPRs [76]. As a result, some IDPs/IDPRs have “block co-polymer” structure, being locally enriched in blocks of similarly charged residues, thereby containing regions of preferentially positively or negatively charged residues that might serve as good candidates for electrostatics-driven LLPTs [56], where the conformational ensembles of such IDPs/IDPRs containing rapidly interconverting and diverse conformers create mean electrostatic fields utilized in polyelectrostatic attraction [77].
(2) Since sequences of many IDPs/IDPRs contain not only clusters of positively or negatively-charged residues, but often also include other sequence repeats of various physico-chemical nature, such repetitive organization can serve as an additional driver of flexible multivalency needed for LLPTs [56].
(3) Since the efficiency of LLPT can be affected by various PTMs [78], and since many IDPs/IDPRs are subjected to different PTMs [38,39], IDPs/IDPRs, with their PTM-controlled conformational variability, are suited well for regulation of PMLO biogenesis [56].
(4) Due to their lack of unique stable structures, IDPs/IDPRs are characterized by high sensitivity to changes in their environment. Such environmental sensitivity and related capability to undergo fast environment-modulated transitions defines the role of IDPs/IDPRs in regulation of LLPTs and PMLOs [56].
(5) The liquid-like character of PMLOs is determined by the lack of unique structure in IDPs/IDPRs involved in LLPTs and the formation of PMLOs and their ability to be engaged in highly dynamic, weak, multivalent interactions [56]. These same properties of IDPs/IDPRs also defines the structural resilience of PMLOs, which are stable entities, despite their lack of membranes, and despite the fact that their constituents are freely exchanged with the environment [56].

### 3.2. Oscillatory Self-Organized Emergent Behavior of Some Bacterial Systems

One of the best-studied examples of a bacterial system with self-organized emergent behavior is given by the Min protein system (MinD, MinC, and MinE) in *Escherichia coli*. Here, Min system is engaged in the spatiotemporal oscillations from pole to pole of the rod-shaped bacterial cells, which is crucially needed for the regulated positioning of the division plane-associated cytokinetic Z ring [79,80]. In vivo spatiotemporal oscillation of Min system is characterized by the wavelength comparable to the size of the *E. coli* cells [81]. This oscillating behavior can be reproduced in vitro by the MinD-driven recruitment of MinE to the bacterial or artificial membranes [82,83,84], generating mesoscale patterns of traveling waves of these Min proteins on the surface of the supported lipid bilayers emerging from the repetitive binding-detaching cycles of proteins to the membrane [85,86].

Another illustration of the oscillatory self-organized emergent behavior in bacteria is given by the members of WAKA protein family (Walker *A* cytomotive ATPase; also knows as ParA). These spatially oscillating proteins are involved in regulation of bacterial development, spatial regulation of cell division, and segregation of chromosomes and plasmids [87,88,89,90]. An illustrative example is given by an interplay between the oscillating ATPase ParA, DNA binding protein ParB, and specific cis-acting DNA regions to which ParB binds that defines the intracellular localization of the *E. coli* plasmids carrying par2 locus [87]. Here, ParA forms oscillating spiral-shaped structures in the presence of ParB and cis-acting DNA regions, but stationary ParA-containing helices extended from one end of the nucleoid to the other are formed in the absence of ParB and DNA [87].

### 3.3. Physiological Liquid-Liquid Phase Transitions in Cells

#### 3.3.1. Liquids in Liquid: Membrane-Less Organelles

The intrinsic disorder-based liquid-liquid phase separation (LLPS, or liquid-liquid phase transitions, LLPTs) in a cell might have different physiological outputs. In fact, LLPTs can lead to the formation of various proteinaceous membrane-less organelles (PMLOs), also known as non-membranous cytoplasmic/nucleoplasmic granules, or intracellular/intranuclear bodies, or cellular/nuclear micro-domains, which are commonly found in cytoplasm and nucleus of various cells [55,56]. In some cases, LLPTs represent a protective mechanism triggered when the cell is exposed to stress. Additionally, it can enable the formation of droplet-like structures that limit the area for molecules to interact, thus increasing the chances of interaction. Behavior like this has been observed during the formation of cytoskeleton components, such as microtubules [91].

Compartmentalization of biomolecules is essential for a cell to carry out its biological functions. This physical separation is achieved using compartments, which are more commonly referred to as organelles. Some organelles achieve separation using a membrane, and these organelles (such as mitochondria, nucleus, Golgi apparatus, endoplasmic reticulum, chloroplasts, etc.) are rather well-known to the scientific community. However, recent studies revealed that there are also proteinaceous membrane-less organelles (PMLOs), which are formed by spontaneous phase-separation into multi-component viscous liquid structures that have cell size–dependent dimensions [69]. These very large (detectable by light microscope), highly dynamic (but stable), and liquid-like assemblages are formed via the intracellular liquid-liquid demixing phase separations [68] and originates due to the colocalization of molecules at high concentrations within a small cellular or nuclear micro-domain [92,93] leading to the LLPTs or the intracellular liquid-liquid demixing phase separation [68,69]. The phase-separation can occur due to changes in the cell environment that trigger molecular supersaturation, such as alterations in concentration of salts or specific small molecules, changes in osmolarity, pH, and/or temperature of the solution, by various PTMs and alternative splicing of the phase-forming proteins, by the binding of these proteins to some definite partners, or by changes in other environmental conditions that affect the protein-protein or protein-nucleic acid interactions [68,69,94,95,96]. The selective partitioning produces a specialized chemical microenvironment that enables specific reactions to occur such as the remodeling of nucleic acids [97]. Since PMLOs are not covered by the membranes, their components are involved in direct contact and exchange with the nucleoplasm or cytoplasm [92,93]. Once formed, PMLOs exhibit hallmark behaviors of liquids, such as fusion upon contact, classical wetting and dripping behaviors, enough surface tension to maintain their spherical shape, and flow in response to shear stresses [98,99,100,101]. Intrinsic density and viscosity of these liquid-droplet phases of the nucleoplasm/cytoplasm/matrix are relatively low being comparable to those of the cytoplasm or nucleoplasm [78,98,99,100,101,102,103,104].

**Figure 2:**
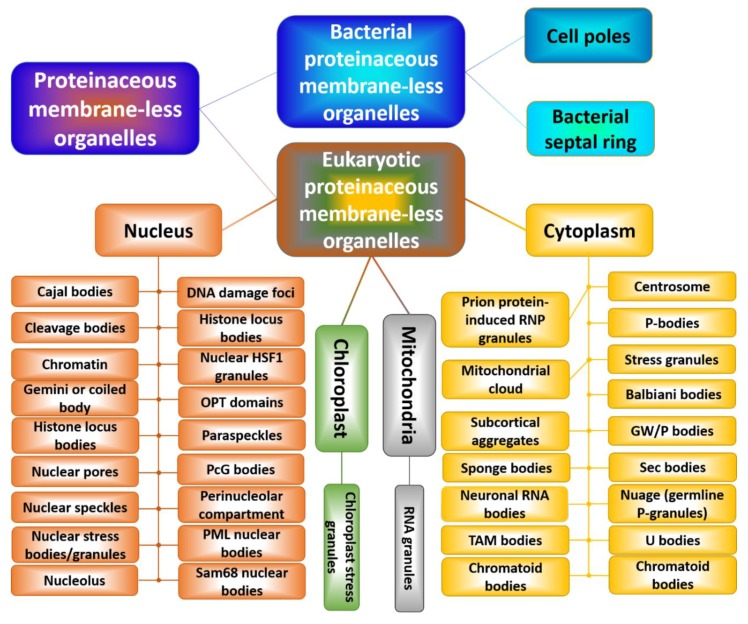
The multitude of cytoplasmic, nuclear, mitochondrial, and chloroplast PMLOs in eukaryotes and bacterial PMLOs. This figure was adopted with permission from Zaslavsky, B.Y., and Uversky, V.N. (2018). In Aqua Veritas: The Indispensable Yet Mostly Ignored Role of Water in Phase Separation and Membrane-less Organelles. Biochemistry 57(17), 2437-2451. Copyright (2018) American Chemical Society.

Figure 2 shows that there is a multitude of membrane-less organelles in the cells that perform a variety of physiological functions [55,56]. In fact, because PMLOs concentrate multiple components, these cellular subdomains serve as important playground for various cellular processes, such as intracellular signaling, mRNA degradation, mRNA transport, ribosome biogenesis, RNA processing, RNP assembly, translational repression, and transcription [75]. PMLOs are also important for specific functional compartmentalization. For example, the nucleus is a membrane bound compartment, but is further partitioned into membrane-less organelles such as the nucleolus and Cajal bodies, just to name a few [53]. The cytoplasm, mitochondria, and chloroplasts are also sites of PMLOs. The cytoplasm contains some PMLOs that are formed because of the cellular stress, such as stress granules. We will discuss some identified roles of the most studied membrane-less organelles, namely the nucleolus, P-granules, and stress granules.

##### Nucleolus

In the nucleus, the largest, and arguably most important, PMLO is the nucleolus. It is the site of ribosomal subunit assembly and research has shown that perturbations in it lead to defects in ribosome assembly and translation [105]. The protein nucleophosmin (NPM1) is the main component of the nucleosome and is required for its formation via liquid-liquid phase-separation [106]. LLPS of NPM1 can influence the direction of assembly of vectorial pre-ribosomal particles within the nucleolus as well as their exit [107]. Once formed, this organelle is dynamic, dissipating once ribosomes are assembled to allow for their export [105]. Aberrations in the formation and dynamics of the nucleoli can lead to an overall decrease in global protein translation [105].

##### Nuclear Pore Complex

Nuclear pore complexes (NPCs) embedded in the nuclear envelope of eukaryotic cells serve as major gates of nuclear transport. NPCs are the largest protein complexes in the cell, possessing a mass of ~125 megaDaltons (MDa) in vertebrates [108] and 66 MDa in yeast [109]. Among 30 nucleoporins (Nups) in yeast NPC, 13 contain phenylalanine-glycine repeats (FG Nups) needed for karyopherin binding and facilitation of the transport of karyopherin-cargo complexes [110]. These FG Nups and particularly their large FG repeat regions were shown to behave as typical IDPs/IDPRs [110]. Furthermore, these low-complexity FG domains were described to phase separate by multivalent cohesion to form a sieve-like selective hydrogel barrier [111,112].

##### Stress Granules

Cytoplasmic PMLOs often form when the cell is exposed to some form of stress. An example of this behavior is given by stress granules (SGs), which are PMLOs containing untranslated messenger ribonucleoprotein (mRNP) formed when the cell is exposed to specific types of stressors [113]. Under certain cellular stresses, translation decreases and the aborted translation initiation complexes are either routed towards translation initiation or degradation [114]. If the stressful insult is mediated by a disruption in protein homeostasis, then the stress-sensing kinase PERK (protein kinase RNA-like endoplasmic reticulum kinase) will phosphorylate eIF2α, and SG formation will ensue [115]. For other types of stress events, various different kinases can be used to phosphorylate eIF2α and induce SG formation. SG assembly is mediated by the prion-like aggregation of TIA1, which causes it to be recruited into SGs in all cell types [113]. Another SG protein component is a phosphorylation dependent endoribonuclease known as RasGAP SH3-binding protein (G3BP) [114]. G3BP interacts with RasGAP in its central domain, where it is dephosphorylated at serine 149, thus recruiting it to SGs [114]. SG assembly is a dynamic process that resolves once the stress inducing insult has been terminated, causing the SGs themselves to dissipate [115]. However, in cases where the insult is too large to overcome, SGs do not clear, and instead the cell switches from a rescue path to a self-induced death pathway [113,115].

##### P-granules

One of the early observations of membrane-less organelles was the discovery of processing bodies (or P-granules), which are germ granules specific to *Caenorhabditis elegans*. P-granules contain RNA granules and RNA binding proteins, mainly PGL1 and PGL3 as well as DEAD-box proteins [116,117]. These granules have been shown to segregate during the development of *Caenorhabditis elegans* germline and exhibit liquid-like behavior [98]. The liquid-like behavior observed involves minimizing surface area, droplet fusions, and flow-like features such as dripping in response to shear stress, which can be explained by the fast internal molecular rearrangements that occur within P granules. Such rapid rearrangements are enabled by multiple weak interactions between RNA molecules and the RNA-binding proteins present in P granules and constitute a general principle of liquid compartments that partition the intracellular space [98].

#### 3.3.2. Reversible Hydrogels

In addition to LLPTs, some proteins can undergo (at least in vitro) reversible liquid-gel phase separation (LGPS), leading to the formation of hydrogels [74], which are not liquid-like PMLOs and cannot flow under steady-state conditions [118,119,120]. Such hydrogels were shown to contain uniformly polymerized amyloid-like fibers, which noticeably are different from the pathological fibrils associated with numerous human diseases [119]. Such hydrogels are highly dynamic systems, being easily and reversibly formed and disassembled in response to some environmental signals, such as addition of specific small molecule or PTMs [119]. Similar to LLPTs, the dynamic LGPS is reversible and depends on multivalent interactions between proteins with LCDs, many of which are known to be intrinsically disordered. Examples of systems undergoing dynamic LGPS include heterotypic polymerization of the LCD of the fused in sarcoma (FUS) RNA-binding protein with RNA [119]; polymerization of mutant FUS forms associated with amyotrophic lateral sclerosis (ALS) [121]; RNA-dependent hydrogel formation of the LCDs of CIRBP, RBM3, hnRNPA1, hnRNPA2, yeast Sup35 protein [119,122], Ewings sarcoma (EWS), and TAF15 proteins [120]; and FG-rich repeat regions of some nucleoporins, such as yeast nucleoporin Nsp1p [118].

#### 3.3.3. Proteinaceous Two-Dimensional Signaling Zones at the Membrane Surface

All the discussed so far LLPTs and LGPS take place in 3-D solutions. However, large (at least micron-sized) two-dimensional protein clusters on the membrane surface can be formed via the dynamic interactions between the multivalent cytoplasmic tails of transmembrane proteins and their multivalent binding partners [123]. The illustrative example of such a system is given by phosphorylated cytoplasmic domain of Nephrin and its intracellular targets, Nck and N-WASP [123]. Importantly, under the appropriate conditions, these three proteins can also form dynamic, micron sized liquid droplets in 3-D solutions [78]. On the other hand, when phosphorylated Nephrin is attached to supported lipid bilayers of DOPC in the presence of Nck and N-WASP, the micron-sized puncta/clusters containing all three proteins are formed on the membranes [123]. These phase-separated two-dimensional (2-D) protein clusters successfully promoted actin filament assembly, and were remodeled themselves by the filament network [123]. These important observations suggested that the multivalent protein interactions and LLPTs can happened both in 3-D and 2-D, and the resulting 2-D micron-scale protein clusters can be responsible for regulation and control of some signaling pathways [123].

### 3.4. Pathological Liquid-Liquid Phase Transitions

For a typical liquid PMLO, there is a specific time and condition window of “safe existence”. This window defines the biogenesis of functional PMLO, whereas outside of this window, the pathological conversion from liquid to solid form within the highly concentrated milieu of PMLO might happen. This pathological conversion can be triggered by extended time of the PMLO existence (or pathological “aging” of PMLOs), or increased concentration of proteins undergoing LLPTs, or aberrant PTMs, or some pathological mutations, or chromosomal translocation [121]. In other words, dysregulated biogenesis of PMLOs and/or loss of their dynamics can serve as important triggers of some pathological conditions.

#### 3.4.1. Pathological “Aging” and Changes in Internal Dynamics of PMLOs

Considered thus far biological LLPTs/LGPS and resulting PMLOs, reversible hydrogels, and 2-D signaling zones clearly have important functions in bacterial and eukaryotic cells. These different phase-separated proteinaceous entities are characterized by physical, dynamic, and mechanical properties that can vary in a broad range. In fact, some of these entities are highly dynamic liquid-like droplets [61,73,75,124,125,126,127,128], whereas others, such as Balbiani bodies, centrosomes, nuclear pores, and amyloid bodies, are much less dynamic “bioreactive gels” with properties ranging from viscous liquids to gels and even to solid-like functional amyloids [129]. One should keep in mind though that these non-dynamic “bioreactive gels” or “biomolecular condensates” are not formed instantaneously. Instead, the very first step of their biogenesis is the formation of dynamic, liquid-like droplets that quickly mature into much less dynamic structures [129]. Furthermore, although many PMLOs (e.g., SGs) are liquid-like in the norm, they are able to mature or age into much less dynamic state, typically coinciding with the formation of fibrous structures [130]. Such maturation leading to the changes in the mechanical and physical properties of cellular bodies can be of biological importance [130]. Of great importance are recent observations that SGs are characterized by the heterogeneous (or biphasic) structures containing a core, where the proteins are more densely packed, and a more diffused shell favoring exchanges of constituents between SGs and the surrounding cytoplasm [115]. Kinetically, these different SG phases are formed at distinct stages of the SG biogenesis, with dense core being assembled at early on in granule assembly [115]. It was also indicated that the SG maturation and time-dependent changes in the dense core of this PMLO can serve as a potential source of insoluble protein aggregates [131].

Therefore, it seems reasonable to hypothesize that at least for some PMLOs, there is a specific “sweet time” of existence, since aberrant biogenesis of PMLOs and their abnormal aging can be accompanied by misfolding and pathological aggregation of PMLO-residing IDPs/IDPRs, being related to the pathogenesis of various human diseases [121,122,132]. In other words, if the stress is sustained, but the cell finds a way to overcome death, it can generate some deleterious effects on the cell later on. In fact, ‘aging’ of SGs can lead to an increase in protein aggregation, and data show that sustained SG formation results in the increased aggregation and cytoplasmic mislocalization of Tar DNA Binding Protein-43 (TDP-43) due to the loss of mobility, both of which are hallmarks in ALS [133]. Recently, it was directly shown that pathological aggregation and fibrillation of low-complexity domain (LCD) of TDP-43 was dramatically accelerated under LLPS conditions, suggesting that aberrant LLPS may contribute to pathogenesis in neurodegenerative disease by promoting pathological TDP-43 aggregation [134]. Liquid droplets formed by the positively charged microtubule-binding domain of intrinsically disordered protein tau were shown to undergo coacervation with negatively charged molecules and this coacervation promoted amyloid fibril formation [135]. In another study, soluble tau was shown to undergo LLPS under cellular conditions, with resulting phase-separated tau droplets rapidly undergoing transition to the gel-like species that eventually matured to amyloid-like fibrils, suggesting that these droplets served as an intermediate toward tau aggregate formation [136]. Finally, many intrinsically disordered RNA-binding proteins (RBPs) possessing LCD domains that are aggregated in patients with different neurodegenerative diseases were found in SGs, suggesting that the dynamics of SGs can be altered by inclusion of such pathology-related proteins [137].

#### 3.4.2. Aberrant PTMs and Pathological Phase Separation

Activity of many proteins is regulated by various PTMs. Since PTMs represent one of the sides of “biological dark matter” (which is composed of biologically important protein species that are not amendable to structural characterization by traditional tools developed to investigate ordered proteins [138]), and since many PTMs occur in IDPRs (that themselves represent another component of the ‘dark matter of biology’), it was indicated that such disorder-centered PTMs constitute the darker side of the biological dark matter [139]. Altogether, by extending the range of structures and physico-chemical properties of amino acids, PTMs play important roles in the increase in the variability and diversity of protein structures and functions [140]. In fact, due to the variability of PTMs, the actual number of chemically modified amino acids typically utilized in protein biosynthesis increases from 20 to more than 140, and as many as 300 different PTMs can be found in proteins [141].

PTMs changes protein structure at many different levels by covalently adding various chemical groups (such as different small molecules, carbohydrates, lipids, and even entire proteins or nucleic acids) to amino acid side chains, or removing various chemical groups, or via enzymatic cleavage of peptide bonds. Since different PTMs can differently affect physicochemical properties of a protein [142], different modifications can graft different functions to the same protein [143]. Although natural variability of PTMs is very broad, these modifications are typically very specific. Many PTMs are catalyzed by special enzymes that recognize particular motifs in target sequences of specific proteins. Some PTMs (e.g., phosphorylation, acetylation, glycosylation, lipidation, methylation, and nitration) are readily reversible due to the concert action of modifying and demodifying enzymes. Such interplay between the conjugating and deconjugating enzymes represents an economical and rapid way of the controlling the protein function. Furthermore, although mutations (which represent another means of changing the chemical properties of a polypeptide chain) can only occur once per position, different forms of PTMs may happen in tandem [144]. Since PTMs represents crucial means for the regulation of protein structure and function, deregulation of PTMs is commonly associated with the development of various pathological conditions [27,139,145]. Therefore, it is not surprising that aberrant PTMs can affect disorder-based LLPTs and PMLOs.

An illustrative example of this concept is given by poly(ADP-ribosylation) (PARylation), one of the PTMs associated with neurodegeneration [146]. PARylation is a reversible enzymatic attachment of multiple NAD-derived ADP-ribose (ADPr) units to target proteins. PARylation is catalyzed by a family of PARP enzymes [147,148], whereas dePARylation is conducted by the hydrolyzing enzyme poly(ADP-ribose) (PAR) glycohydrolase (PARG) [149,150]. Furthermore, some proteins are capable of non-covalent PAR-binding [151]. In addition to numerous physiological roles of PARylation that range from gene expression to DNA repair, mitochondrial biogenesis, neuroinflammation, and regulation of a variety of signaling pathways inducing different forms of cell death, alterations in this PTM were associated with aberrant LLPTs and pathological aggregation of several proteins, such as α-synuclein, TDP-43, and heterogeneous nuclear ribonucleoprotein A1 (hnRNPA1) [146] associated with Alzheimer’s disease (AD), Parkinson’s disease (PD), Huntington disease (HD), and amyotrophic lateral sclerosis (ALS). Since PAR is characterized by a multivalent anionic polymeric structure resembling nucleic acids, and since many neurodegeneration-related proteins are RBPs that also contain the PAR-binding motifs (PBM), increased levels of PAR can directly influence amyloid aggregation of some PBM-containing proteins or modulate LLPTs in other pathology-associated RBPs, such as TDP-43 and hnRNPA1 [152], or stimulate association of some of these RBPs with SGs [146]. Furthermore, the picture is further complicated by the fact that hnRNPA1 can be PARyated, and this PTM facilitates the LLPT of hnRNPA1 alone, and also stimulates the co-LLPT of TDP-43 and hnRNPA1 [153].

An AD-related intrinsically disordered microtubule-associated protein tau is known to undergo LLPS [135], the efficiency of which can be affected by various mutations and PTMs, such as truncation, hyperphosphorylation, and hyperacetylation [135,154]. Importantly, these different factors differently affect the LLPS behavior of tau, with truncation, mutation, and hyperphosphorylation enhancing LLPS and aggregation [135], and with hyperacetylation disfavoring LLPS and inhibiting the heparin-induced aggregation of this protein [154]. Although the LCD of RNA-binding protein hnRNPA2B1 (heterogeneous nuclear ribonucleoprotein A2B1) can undergo LLPT and promotes hnRNPA2B1- TDP-43 co-phase separation, the arginine methylation of this domain reduces the efficiency of hnRNPA2 phase separation and inhibited co-phase separation of this protein with TDP-43 [155].

#### 3.4.3. Mutations and Pathological Phase Separation

Biogenesis of PMLOs can be affected by pathological mutations in proteins either undergoing LLPTs or proteins involved in PMLO regulation. For example, different properties of SGs, such as their number, mean size, lifespan, and internal dynamics, as well as the SG capability to control stress suppression are all affected by TDP-43 with ALS-related point mutations [156,157]. Similarly, biogenesis of SGs (namely, kinetics of their assembly and disassembly) is affected by ALS-related point mutations in FUS, which become incorporated into SGs [158]. Similarly, heterogeneous nuclear ribonucleoproteins (hnRNPs) A2B1 and A1 (hnRNPA2B1 and hnRNPA1) with ALS-related point mutations in their prion-like domains noticeably alter SG biogenesis and dynamics, being excessively incorporated into this PMLO [159]. On the other hand, ALS-related point mutations in T-cell-restricted intracellular antigen-1 (TIA1) not only impacted the SG dynamics but also promoted the accumulation of stable SGs that contained TDP-43 [133]. Systematic analysis of several familial ALS-related point mutations in the proteasomal shuttle factor UBQLN2 mostly affecting the proline-rich (Pxx) region of this protein revealed that the UBQLN2 LLPT was differently affected by these ALS-linked Pxx mutations [160]. This differential effect was dependent on the type and sequence position of a given amino acid substitution, suggesting that ALS-linked Pxx mutations altered physical properties of UBQLN2, modified the in vivo behavior of this protein, and contributed to the aberrant morphology and dynamics of SGs, eventually resulting in the appearance of ALS specific inclusions [160].

It was shown that the unnatural dipeptide repeat (DPR) proteins (poly(glycine-alanine), polyGA; poly(glycine-arginine), polyGR; poly(proline-alanine), polyPA; poly(proline-arginine), polyPR; and poly(glycine-proline), polyGP) generated as a result of the hexanucleotide (GGGGCC) repeat expansion in the gene chromosome 9 open reading frame 72 (*C9ORF72*), which is considered now as the most common cause of ALS and frontotemporal dementia (FTD), were able to alter the liquid-like state of PMLOs [161]. Furthermore, arginine-rich DPRs (polyGR and polyPR) were shown to undergo LLPS themselves and were able to efficiently induce phase separation of a large set of proteins related to the RNA metabolism and SG biogenesis [162].

#### 3.4.4. Chromosomal Translocation and Pathological Phase Separation

NUP98 is one of the NPC proteins that contain FG-repeat domains. Physiologically, NUP98 plays an important role in assembly and/or maintenance of NPC and in the bidirectional transport across the NPC [111]. Wild-type NUP98 primarily localizes to the NPC, with its intrinsically disordered FG-repeat domain (~500 residues) filling the central pore. Although most NUP98 localizes to the NPC, a small portion of the protein resides in the nucleoplasm, being localized to specific sites on chromatin, where it enhances transcription of genes involved in cell cycle regulation and cell differentiation [163,164,165]. The intrinsically disordered N-terminally located FG-repeat domains of Nup98 from different species (amoebas, ciliates, excavates, fungi, insects, lancelets, mammals, nematodes, and plants) were shown to undergo fast and spontaneous phase-separation from dilute aqueous solutions into characteristic ‘FG particles’ or gel-like bodies in vitro [111]. These domains also phase separate into large, spherical puncta in the nuclei of cells [163,166]. In adult and pediatric hematological malignancies (such as acute myeloid leukemia (AML) and acute erythroid leukemia (AEL)), NUP98 is commonly fused to various proteins associated with gene regulation [164,166,167]. The resulting NUP98 fusion oncogenes typically encode fusion oncoproteins (FOs) containing the N-terminal FG-repeat domain of NUP98 fused in frame with a C-terminal homeobox DNA-binding (i.e., HOXA9, HOXD13) or histone binding or modifying (i.e., KDM5A, NSD1, PHF23) domains of corresponding gene regulating proteins [164,166,167]. Importantly, although large puncta formed by the NUP95 and its FG-repeat domain are not related to hematopoietic cell transformation and leukemia development [168], the NUP98-based FOs form, through phase separation, many chromatin-associated, sub-micron-sized puncta that are associated with aberrant gene transcription, hematopoietic cell transformation and pathogenesis of leukemia [166]. These FOs, found within small, liquid-like nuclear puncta, might serve as critical transcriptional regulators [169,170,171,172], driving co-localization of distal chromatin sites and organization of the transcriptional machinery for coordinated expression of multiple genes [127]. On a more general note, these chromatin remodeling activities of phase separated NUP98-based FOs represent a pathological counterpart of physiological roles of chromatin-associated liquid droplets, where phase separation of heterochromatin domains with heterochromatin protein 1 (HP1α) coordinates chromatin compaction and gene silencing [173,174], and phase separation-generated nuclear puncta function as transcription centers containing transcription factors and RNA polymerase II (RNA Pol II) [127,169,170,171,172].

Translocations leading to the fusion of RNA-binding domains of FUS, Ewings sarcoma breakpoint region 1 (EWSR1), and TATA-binding protein-associated factor 15 (TAF15) proteins, which are collectively known as FET (FUS/EWS/TAF15) proteins [175], with the homeobox, zinc finger, ETS, or leucine zipper families DNA binding domains found in various transcription regulators (such as different ETS transcription factors (e.g., friend leukemia integration 1 transcription factor, FLI1), C/EBP-homologous protein (CHOP), transforming protein ERG, etc.), generate chimeric FOs associated with pathogenesis of various forms of cancer, including Ewing’s family tumors [176,177,178]. LCDs of FET proteins were shown to undergo phase separation leading to the formation of hydrogels, which were able to interact with C-terminal domain (CTD) of RNA polymerase II in a CTD phosphorylation-dependent manner [120]. In Ewing sarcoma, EWSR1-FLI1 fusion protein phase-separates (via the prion-like domain of EWS) and specifically targets the BRG1/BRM-associated factor (BAF) chromatin remodeling complex to tumor-specific enhancers and contributes to target gene activation, thereby eliciting the aberrant transcriptional programs underlying Ewing’s sarcoma [172,179].

## 4. Water Side of LLPTs and PMLOs

Finally, one more important point should be addressed here, namely the (mostly ignored) role of water in LLPTs and biogenesis of PMLOs. There is no doubt that protein structure and function cannot be considered apart from the consideration of the most universal natural solvent, water. As a matter of fact, water determines how a given protein would look like and what it will do. In other words, structure and function of a protein are determined not only by its amino acid sequence, but also by its environment, where water plays a crucial role. On the other hand, various physicochemical properties of aqueous solutions (such as solubility of different solutes, dielectric properties, surface tension, water activity, osmotic coefficient) of different compounds differ from properties of pure water and are commonly concentration- and compound nature-dependent [180,181]. Nonionic polymers, typically used for in vitro modeling of the conditions of macromolecular crowding [182,183], Hofmeister series of sodium salts [184], osmolytes [185], and some proteins [186,187,188], induce changes in the solvent features of water (such as solvent dipolarity/polarizability (π*), its hydrogen bond acceptor basicity (β), and hydrogen bond donor acidity (α)) in the compound natures and concentration specific manner. In agreement with these observations, recent spectroscopic analysis using infrared and polarized Raman spectroscopies revealed that water structure is changed by the addition of water soluble polymers, such as polyethylene glycol (PEG-4000) and Ucon-4000 [189]. Furthermore, a recent study showed that the effects of nonionic polymers and/or osmolytes on stability of proteins and nucleic acids may be quantitatively described by the effects of these polymers and osmolytes on the aforementioned solvent features of water [182]. It was hypothesized that proteins and nucleic acids undergoing LLPTs also may alter solvent properties of aqueous media [180]. Due to their extended structures defined by the peculiar amino acid sequences, IDPs/IDPRs interact with water differently in comparison with interaction of globular proteins with water [190,191,192,193,194,195]. This suggests that the enhanced capability of IDPs/IDPRs to interact with water and change its solvent properties might contribute to their common involvement in PMLO formation. Therefore, changed solvent properties of water in the presence of IDPs/IDPRs might represent a driving force for the biological LLPS and contribute to the biogenesis of various PMLOs [180,181].

## 5. Conclusions

Materials presented in this review shows that IDPs/IDPRs are characterized by specific emerging behavior, being able to form “running waves” (as in the case of Min protein system) or show oscillatory self-organization (e.g., bacterial WAKA proteins). IDPs and hybrid proteins containing ordered domains and IDPRs can undergo LLPTs or LGPTs, generating various PMLOs, transcription centers, responsive hydrogels, and 2-D signaling zones at the membrane surface. These self-organized cellular compartments have numerous biological roles and are crucial for normal physiology of the cell. On the other hand, aberrant LLPTs and LGPTs, as well as “aged” PMLOs with distorted dynamics are serious “troublemakers”, associated with various maladies, such as neurodegenerative diseases and cancers.

LLPTs are driven by weak stochastic multivalent interactions originating from the structural/sequence modularity and multivalency, features that are commonly found in IDPs/IDPRs [53,55,56]. LLPTs are completely reversible, fast, highly controlled, and strongly condition-dependent. Phase separation takes place only when specific conditions, such as critical protein and RNA/DNA concentration [78], and/or critical level of PTMs, and/or critical temperature, pH, osmolarity, or other environmental conditions affecting protein-protein interactions [55,56,69,74,94,95] are reached, whereas complete disintegration of the condensed phase is triggered by leaving this “comfort zone”. In other words, LLPT happen when a critical threshold is crossed, giving rise to conditions suitable for phase separation [74,95].

Aberrant LLPTs, distorted PMLO biogenesis, and lost/decreased PMLO internal dynamics are at the heart of the pathological liquid-liquid phase transition concept. In fact, in addition to the “comfort zone” of conditions promoting LLPTs, PMLOs are characterized by a specific time and condition window of “safe existence”. Many PMLOs should be present for a given time in a given place at a given moment. Physiologically undesired extension of the time of their safe existence might result in pathological “aging” of PMLOs that would trigger development of some pathological conditions (e.g., various neurodegenerative diseases, where aged PMLOs serve as a source of pathological aggregation of related amyloidogenic proteins). Other factors causing spoiled LLPTs and pathological conversions of PMLO include increased concentration of proteins undergoing LLPTs, or aberrant PTMs, or some pathological mutations, or chromosomal translocation.
